# Anxiolytic activity of pyridoindole derivatives SMe1EC2 and SMe1M2: behavioral analysis using rat model

**DOI:** 10.2478/v10102-011-0032-8

**Published:** 2011-12

**Authors:** Natália Sedláčková, Veronika Ponechalová, Eduard Ujházy, Michal Dubovický, Mojmír Mach

**Affiliations:** Institute of Experimental Pharmacology & Toxicology, Slovak Academy of Sciences, SK-84104 Bratislava, Slovakia

**Keywords:** behavioral analysis, anxiety, depression, pyridoindole, rat

## Abstract

Anxiety and mood disorders have become very significant affections in the last decades. According to WHO at least one mental disease occurred per year in 27% of EU inhabitants (more than 82 mil. people). It is estimated that by 2020, depression will be the main cause of morbidity in the developed countries. These circumstances call for research for new prospective drugs with anxiolytic and antidepressive properties exhibiting no toxicity and withdrawal effect and possessing beneficial properties, like antioxidant and/or neuroprotective effects. The aim of this study was to obtain information about psychopharmacological properties of pyridoindole derivatives SMe1EC2 and SMe1M2, using non-invasive behavioral methods in rats.

The battery of ethological tests (open field, elevated plus-maze, light/dark box exploration, forced swim test) was used to obtain information about anxiolytic and antidepressant activity of the pyridoindole derivatives. The substances were administered intraperitoneally 30 minutes before the tests at doses of 1, 10 and 25 mg/kg.

In the behavioral tests, SMe1EC2 was found to exert anxiolytic activity in elevated plus maze with no affection of locomotor activity. The highest dose of SMe1M2 increased the time spent in the lit part of the Light/Dark box, however this result was influenced by inhibition of motor activity of the rats. Similar findings were observed also in elevated plus-maze, although these results were not statistically significant.

In conclusion, from the results of our study it is evident that both pyridoindoles acted on the CNS. In the highest dose, SMe1M2 was found to possess rather sedative than anxiolytic or antidepressant activity.

## Introduction

Psychiatric disorders have accompanied mankind from the beginning of its existence. Compared to the pass, the current hectic lifestyle is far more complicated, affecting the social environment. Fast, unpredicted changes cause increased pressure, requiring repeated adjustment. Sometimes these adaptations trigger a cascade of unpredicted events, which may lead to self-destructive behavior or even suicide.

Anxiety is defined as a state of unwarranted or inappropriate worry, often accompanied by restlessness, tension, distraction, irritability, and sleep disturbances, resulting in somatic manifestations of anxiety, including shortness of breath, sweating, nausea, rapid heartbeat, and elevated blood pressure (Sandford *et al.*, 2000).

Generalized anxiety disorder (GAD) is the most common of the anxiety disorders, with a lifetime prevalence of approximately 5% (Wittchen & Hoyer, 2001). More than 27% of adult Europeans are estimated to experience at least one form of mental ill health during any one year (Wittchen & Jacobi, 2005). The most common forms of mental ill health in the EU are anxiety disorders and depression. By the year 2020, depression is expected to be the highest ranking cause of disease in the developed world (WHO, 2001). Currently, in the EU, some 58,000 citizens die by suicide every year, more than the annual deaths from road traffic accidents, homicide, or HIV/AIDS (European Commission, 2005).

Introduced over 40 years ago, benzodiazepines (BZs) quickly became the most widely used psychotropic drugs. However in recent years, attitudes toward these compounds have greatly changed, and growing awareness and concern about dependence liability, withdrawal phenomena, and short- and long-term side effects have made the long-term use of these compounds into question able (Basile *et al.*, 2004). The search has begun for compounds chemically unrelated to BZs with more specific therapeutic actions and without concomitant unwanted effects. Pyridoindol compounds such as [6-Fluoro-9-methyl-2-phenyl-4-(pyrrolidin-1-yl-carbonyl)-2,9-dihydro-1*H*-pyridol[3,4-b]indol-1-one] have proved to be full agonists (in electrophysiological assays) at GABAA2 receptors, and may have the clinical potential as anxiolytics and muscle relaxants (Licata *et al.*, 2005).

Both pyridoindoles SMe1EC2 and SMe1M2 were derived from the pyridoindol stobadine (STO) [(–)-cis-2,8-dimethyl-2,3,4,4a,5,9b-hexahydro-1*H*-pyrido[4,3-b]indole] is derived from the gamma-carboline antidepressant and neuroleptic drug Carbidine® as its active (–)-enantiomer. Preclinical safety evaluation of STO was performed in rats (Gajdošíkova *et al.*, 1995), beagle dogs (Majerčík *et al.*, 1984) and *in vitro* in human fibroblastoid cells (Chalupa *et al.*, 1993) and no adverse effects were detected. It crosses readily the blood brain barrier and has cardioprotective, neuroprotective and antihypoxic properties (Horákova and Štolc, 1998). Moreover, long-term administration of STO resulted in increased levels of dopamine and serotonin (Dubovický *et al.*, 1997). Based on STO, new pyridoindole derivatives with improved pharmacodynamic and toxicity profiles have been developed on applying molecular design, synthesis and adequate tests. A total of >70 derivatives have so far been prepared. These derivatives have low toxicity (LD_50_ after p.o. administration>2000 mg/kg – Štolc *et al.*, 2008).

This study was the first experiment with the pyridoindole SMe1M2, yet for SMe1EC2, better knowledge on toxicity, antioxidant properties and neuroprotection is available. Ujházy *et al.* (2008; 2011) did not find any maternal, embryofetal neurobehavioral toxicity in their safety studies. A positive neuroprotective effect was also observed in 2-month-old male rats by improved recovery of neuronal function after transient hypoxia/hypoglycemia in reoxygenation, as well as by morphological changes manifested as reduced edema extent in the treated hippocampus under ischemia in vitro (Gáspárová *et al.*, 2009).

The aim of this study was to assess psychopharmacological properties of the pyridoindole derivatives SMe1EC2 and SMe1M2, using non-invasive behavioral methods in rats.

## Material and methods

### Animals

Eighty 3–4 months-old adult male Wistar rats (Department of Toxicology and Breeding Station at Dobrá Voda; Slovakia) weighing approximately 250±50g were used for the study. The rats were acclimatized to the animal housing facility for 10 days prior to experimental procedures. The animals were housed in polypropylene cages measuring 35×55×20cm, covered by a stainless steel netted lid, placed in groups of 4. The bedding material consisted of wood chips. The animals had free access to standard pelleted diet (KKZ rat/mouse, registration number SK 100089) and water. Ambient temperature and relative humidity were maintained at approximately 22±2°C and 55±10%, respectively. The animal room was illuminated by artificial light from fluorescent tubes on a 12-h light/dark cycle, lit during the day from 7 a.m.

All procedures were performed in compliance with the Principles of Laboratory Animal Care issued by the Ethical Committee of the Institute of Experimental Pharmacology and Toxicology, Slovak Academy of Sciences and by the State Veterinary and Food Administration of the Slovak Republic.

### Drugs

Pyridoindol derivatives (code names SMe1EC2 and SMe1M2) were prepared at the Institute of Experimental Pharmacology and Toxicology, Slovak Academy of Sciences. The substances tested were dissolved in saline at a constant dosage volume 0.1ml/100g body weight. Each rat was treated intraperitoneally at a single dose of 1, 10 or 25 mg/kg 30 minutes before the behavioral test. Controls received vehicle.

### Experimental procedures

The animals were assigned to groups according to their basal motor activity measured using an open field (base 42×42cm, height 40cm) equipped with Actitrack (Panlab, S. L., Spain). The apparatus software recorded locomotor activity of the animal by registering the beam interruptions caused by movements of the body. All behavioral experiments were performed 30 min after i.p. injection of the drugs.

#### Open field test

The standard open field test is commonly used to assess locomotor, exploratory and anxiety-like behavior in laboratory animals (rats/mice). This test is particularly useful for evaluation of the anxiolytic and anxiogenic effects of drugs, locomotor responses to drugs as well as behavioral responses to novelty.

The open field used in this experiment consisted of a 60×40cm plastic apparatus, closed with 25cm-high walls. The apparatus was kept in a room under dim light and the tests were undertaken during the light-on phases of the cycle, between 9 a.m. and 1 p.m. In order to eliminate any olfactory cues, the apparatus was cleaned with mild detergent after each individual test. Each session was started by placing the rat in the central area and lasted 5 min. Two animals were tested simultaneously and the rats′ movements were tracked with two digital cameras and the movies were analyzed by computer software ANY-Maze (Stoelting Europe, Ireland).

#### Elevated plus maze

The standard elevated plus maze test is commonly used to assess anxiety-like behavior in laboratory animals (rats/mice). The maze is a cross-shaped elevated maze, with two open arms and two closed arms. The elevated plus maze task approaches the conflict between the innate fear that rodents have in open areas versus their desire to explore new environment. Security is provided by the closed arms whereas the open arms offer exploratory value. When anxious, the natural tendency of rodents is to prefer enclosed dark spaces to opened brightly lit spaces. In this context, anxiety-related behavior is measured by the degree to which the rodent avoids the unenclosed arms of the maze.

All parts of the apparatus were made of dark polyvinyl plastic. The arms of the maze were 50 cm above the floor, 50 cm long and 10 cm wide. Two animals were tested simultaneously. The rats’ movements were tracked with two digital cameras and the movies were analyzed by computer software ANY-Maze (Stoelting Europe, Ireland). Lighting was provided by a string of rope lights attached to the underside of the open arms of the maze. Each session was started by placing the rat in the central area facing the open arms of the maze and lasted 5 min. Between trials, the maze was wiped with a mild detergent.

#### Light/Dark Test

The test apparatus consisted of white plastic arena (60×40cm) with black plastic box (40×20×40cm, opening 10×8cm) inserted in the arena. Anincandescent light bulb fixed 1 m above the arena provided the illumination so that the light intensity in the center of the illuminated box was 200 lux. At the beginning of the experiment, a rat was placed in the illuminated box, facing the opening. The apparatus was equipped with digital camera and the movies were analyzed by computer software ANY-Maze (Stoelting Europe, Ireland). The following parameters were recorded during a 5-min period: (a) time spent by rats in the lit box; (b) time spent by rats in the dark box; (c) attempt at entry into the lit box followed by avoidance responses.

### Statistical analysis

The data were analyzed by means of one-way ANOVA followed by LSD *post hoc* test. The data are expressed as mean±S.E.M. The significance limit of *p<*0.05 was considered statistically significant.

## Results

### SMe1EC2 anxiolytic properties

**Figure 1:**
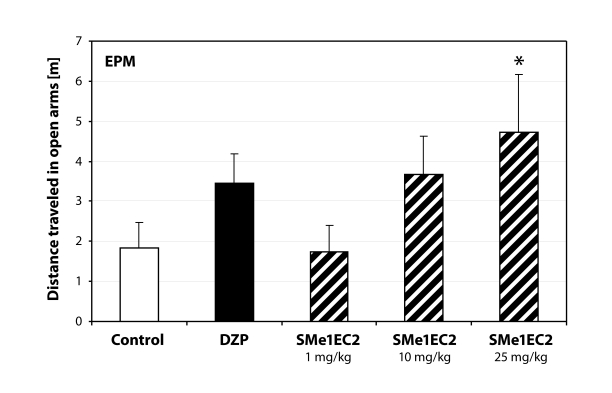
Anti-anxiety behavior manifested in rats treated with highest dose of SMe1EC2. DZP – diazepam 2.5 mg/kg; *p<0.05 – significantly different compared to controls (ANOVA).

Fisher LSD *post-hoc* analysis revealed that only the highest dose increased the activity of rats in the open arms of the elevated plus maze (*p<*0.05) (Figure 1). We also noticed an increasing trend of time spent in the open arms depending on the dose administered.

**Figure 2:**
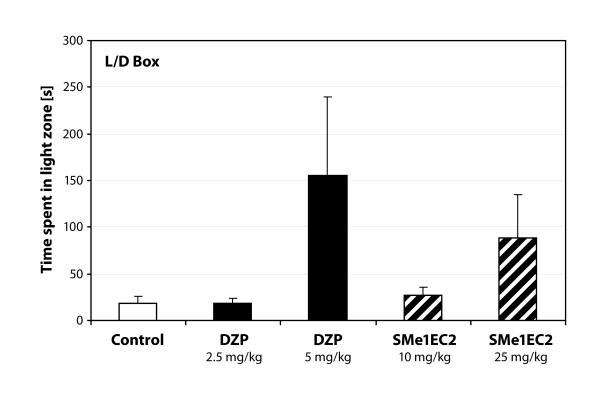
Time spent in the lit part of the Light/Dark Box. DZP – diazepam (positive control).

We discovered that animals tended to be less anxious also in the light/dark test only after treatment with highest dose. However, this result was not statistically significant (Figure 2). No differences were seen in locomotor activity tested in open field.

### SMe1M2 anxiolytic properties

**Figure 3:**
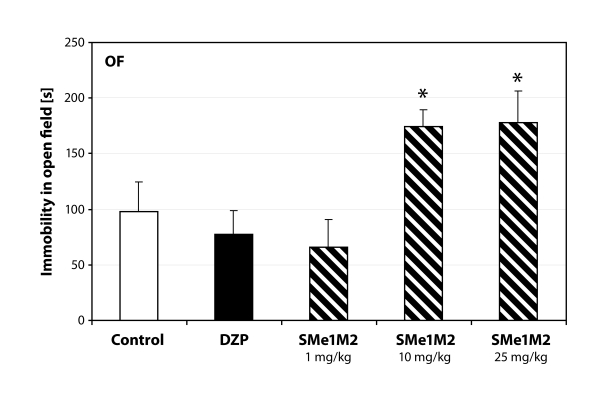
Sedative behavior manifested in rats treated with SMe1M2. DZP – diazepam 2.5 mg/kg; *p<0.05 – significantly different compared to controls (ANOVA).

**Figure 4:**
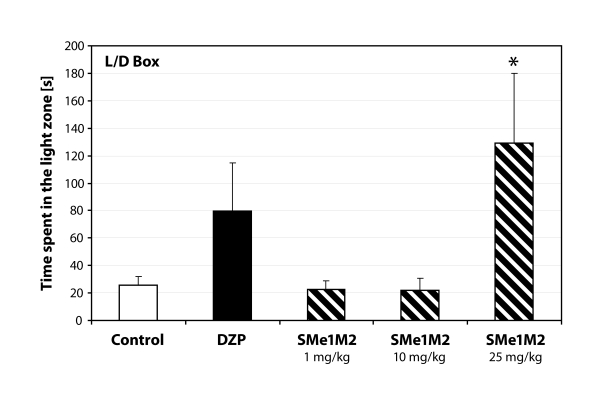
Anti-anxiety behavior manifested in rats treated with highest dose of SMe1EC2. DZP – diazepam 5 mg/kg; *p<0.05 – significantly different compared to controls (ANOVA).

**Figure 5:**
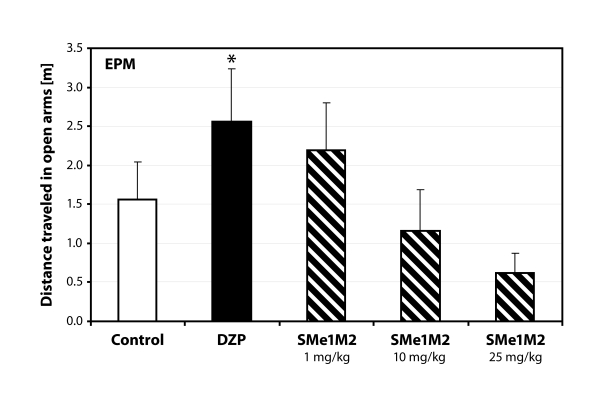
Dose-dependent inhibition of locomotor activity after treatment with SMe1M2. DZP – diazepam 2.5 mg/kg; *p<0.05 – significantly different compared to controls (ANOVA).

Administration of SMe1M2 increased the immobility in the open field (*p=*0.05; Figure 3). Similarly were influenced also the following parameters tested. Animals receiving the highest dose of SMe1M2 spent significantly more time in the lit part of the light/dark box (*p<*0.05; Figure 4) and the activity in the open arms was also influenced in a dose-dependent manner (Figure 5).

## Discussion

Problems of mental health are still tabooed. Cancer research is predominant and well supported while mental-health research is focused mainly on neurodegenerative diseases such as Alzheimer's, rather than on earlier-onset conditions that can undermine peoples’ entire lives, such as depressive disorders. It is startling that approximately one mental disease has found to occurr per year in 27% of EU inhabitants, representing more than 82 million people. WHO (2001) estimated that by 2020 depression would be the main cause of morbidity in the developed countries.

The aim of this study was to assess psychopharmacological properties of two pyridoindole derivatives SMe1EC2 and SMe1M2 synthesized at the Institute of Experimental Pharmacology and Toxicology. We used a non-invasive ethological approach of behavioral analysis. Both derivatives originate from stobadine (STO), whose molecule was studied in detail and no adverse effects were observed (Gajdošíková *et al.*, 1995; Balonová *et al.*, 1991; Dubovický *et al.*, 1999). STO is an optic stereoisomer of the neuroleptic drug γ-carboline Carbidine^®^ (Barkov, 1973). Our results showed possible anxiolytic properties of SMe1EC2. The substance did not influence the motor activity at the doses tested and the elevated plus maze test revealed dose-dependent decrease of anxiety behavior after treatment with SMe1EC2. The highest dose increased the activity of rats in the open arms. The activity of rats treated with the dose of 1mg/kg was comparable to that of control rats, however the time spent in the intersection was increased, indicating an anti-anxiety type of behavior. Similar results were seen in the light/dark box.

The second derivative, SMe1M2, caused a dose-dependent decrease in motor activity seen in open field as well as elevated plus maze. The effect of the highest dose (25mg/kg) was statistically significant. We did not observe anti-anxiety behavior similar to that of SMe1EC2. Increased immobility could be responsible for false positive results in the light/dark box test. Animals receiving the highest dose spent significantly more time in the lit part of the box compared to controls.

Even though benzodiazepines are relatively safe drugs and are widely used in the treatment of anxiety, they may produce untoward side effects such as sedation, memory impairment, tolerance, and physical dependence (Griebel *et al.*, 2001).

Pharmacological effects of benzodiazepines are produced through positive allosteric modulation of the action of GABA at ionotropic GABA_A_ receptors (Braestrup & Squires, 1977; Mohler & Okada, 1977; Barnard *et al.*, 1998). The classical benzodiazepines interact indiscriminately with heterogenous GABA_A_ receptor subtypes, and possess numerous useful and also unwanted pharmacological actions. Researchers therefore search for compounds chemically unrelated to the benzodiazepines, with eliminated unwanted side effects and more specific therapeutic actions.

The clinically used benzodiazepines are full agonists. Attempts have been made to develop compounds which are anxioselective in that they retain the anxiolytic properties of the full agonist, but have reduced sedation and dependence (withdrawal) liabilities. Such compounds may interact with all four (i.e. α1-, α2-, α3- and α5-containing) GABA_A_ receptor subtypes and have partial rather than full agonist efficacies. Examples of nonselective partial agonists include bretazenil, imidazenil, FG 8205, abecarnil, NS 2710, pagoclone, RWJ-51204 and (S)-desmethylzopiclone (Atack, 2003). Recently there has been more evidence pointing toward pyridoindole derivatives as possible replacement of benzodiazepines in treatment of anxiety disorders. Behavioral studies in rodents showed that SL651498 ([6-Fluoro-9-metyl-2-fenyl-4-(pyrolidin-1-yl-karbonyl)-2,9-dihydro-1*H*-pyridol[3,4-b]indol-1-one]) had anxiolytic-like activity similar to that of diazepam (Griebel *et al.* 2001, 2003). The SL651498 induced muscle weakness, ataxia, or sedation occurred at doses much higher than those producing anxiolytic-like activity (Griebel *et al.*, 2001).

Similar to the findings of Griebel *et al.* (2001), we observed anxiolytic properties of our pyridoindole derivative SMe1EC2. Moreover, our chosen dose range did not induce motor activity depression and immobility. SMe1M2 produced a greater inhibitory effect on overall motor activity with significant increase in immobility. Several authors reported that compared to benzodiazepines non-selective partial agonists of GABA_A_ receptors e.g. bretazenil, imedazenil and Y-23684, possessed better anxiolytic properties and did not induce such prominent inhibition of motor activity. It is estimated that the sedative and anxiolytic action of GABA_A_ receptors are mediated via α1 and α2 GABA_A_ receptors (Crestani *et al.*, 2000; Löw *et al.*, 2000; Rudolph *et al.*, 1999). These autors have suggested that the α1-subunit is responsible for sedative effects and the α2-subunit for their anxiolytic properties.

Our results showed that the pyridoindoles SMe1EC2 and SMe1M2 synthesized at the Institute of Experimental Pharmacology and Toxicology influenced the subtle function of the CNS controlling anxiety and/or alertness of animals. It is evident that SMe1M2 affected locomotor activity and the doses used induced sedative-like behavior. This study was the first experiment with SMe1M2 and the doses were chosen according to a previous study of pyridoindole derivatives. With optimalization of the dose it is still to be elucidated if this pyridoindole possesses similar anxiolytic properties as SMe1EC2. Taking into account that these derivatives have excellent antioxidant and neuroprotective properties and exhibit low toxicity, they might represent new directions in the treatment of anxiety disorders.
